# The pH Levels of Different Methamphetamine Drug Samples on the Street Market in Cape Town

**DOI:** 10.5402/2011/974768

**Published:** 2011-06-26

**Authors:** Sias R. Grobler, Usuf Chikte, Jaco Westraat

**Affiliations:** Oral & Dental Research Institute, Faculty of Dentistry, University of the Western Cape, Tygerberg, Cape Town 7505, South Africa

## Abstract

The purpose of this study was to determine the pH levels of 29 different samples of methamphetamine on the street market in Cape Town. The sample was dissolved in water and the pH of each sample determined. The pH levels varied from 3.02 to 7.03 with an average of 5.0. Seventy-two percent (21) of the samples had a pH level below the saliva “critical pH point of 5.6” and therefore should cause significant damage to enamel, especially in hyposalivation subjects without a saliva flow. However, about 26% of the samples had a pH level close to the neutral point and should cause minor damage to enamel. To lessen enamel damage, subjects should exercise good oral hygiene practice, rinse with a fluoride-containing mouth rinse, drink artificially sweetened drinks, and eat cheese. It is concluded that most of the methamphetamine samples have a low enough pH to cause direct damage to enamel especially in hyposalivation subjects.

## 1. Introduction

Methamphetamine was first synthesized from ephedrine in Japan in 1893 by the chemist Nagai [1]. Methamphetamine is approved by the US Food and Drug Administration (FDA) for the treatment of attentiondeficit hyperactivity disorder and exogenous obesity and is marketed in the USA and Canada under the trademark name Desoxyn [2].

Methamphetamine (or 2-n-methyl-1-phenyl-propan-2-amine or metamfetamine) is a highly addictive, psychostimulant, and sympathomimetic drug, illicitly used in many countries [3]. On the street, it is commonly found as a colourless, crystalline solid, and frequently adulterated with chemicals that were used to synthesize it. It is also known as “crystal meth,” “ice,” “glass,” “tina,” “christine,” “yaba,” “tik,” “crazy medicine,” and others [4]. The product can be eaten, ground and snorted, dissolved and injected or drank or smoked. Chronic abuse of methamphetamine results in hyposalivation, rampant caries, bad taste, bruxism, and muscle trismus [4–8]. 

The distinctive features of dental caries in chronic methamphetamine users are known as “meth mouth”. A photo (enamel erosion) was included at the end of this paper to demonstrate a typical “meth mouth”. “Meth mouth” is probably caused by a combination of factors which resulted from the psychological and physiological changes as a result of the use of the drug. These factors include hyposalivation, poor oral hygiene, consumption of soft drinks high in carbohydrates, and bruxism. Some also speculated about the acidic nature of the drug as an important contributing effect [9], while others believe that the neglectance of oral hygiene is the main culprit in the development of caries [10, 11]. The fact that the methamphetamine users tend to consume large volumes of carbohydrates in the absence of the buffering effect of saliva and no oral hygiene practices is a further contributing effect to the destruction of the teeth because of the formation of acids through plaque fermentation. This effect is further intensified by the fact that the duration of the drugs effect is ~12 hours whereas it is one hour for cocaine [9].

 There are many ways to prepare methamphetamine of which the Leuckart route seems to be the most favorable [10]. The chemical formula for methamphetamine is 
(1)
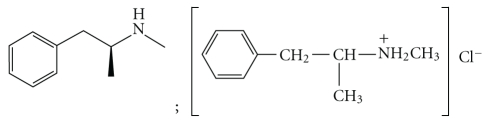

It is believed that the number and combination of impurities found in the final product is a sort of fingerprint of the type of methodology used in the manufacturing thereof [10]. 

The analysis of the illegal drug is mostly done through the use of ion chromatography, inductively coupled plasma mass spectrometry (ICP-MS), or a combination of ion chromatography and ICP-MS [9]. The synthesis of methamphetamine mostly includes, at some or other stage, an acidic medium of some kind like the most widely used reductive amination of benzyl methyl ketone [10] or the Leuckart reaction [11]. However, other less known ways of synthesis are also known but seldom used [12, 13]. Impurities reported in the preparation of methamphetamine through the reductive animation process are benzyl methyl ketone, amphetamine, 1-phenyl-2-propanol, N,N-dimethylamphetamine, and dibenzylketone [14, 15].

Methamphetamine is actually the hydrochloric (HCl) salt of methamphetamine [16] because the synthesis includes the pumping of hydrochloric acid gas through the solution to crystallize the drug. If the crystals are then not purified, the hydrochloric gas is also trapped in pockets which will then dissolve and acidify the oral cavity. Furthermore, when the drug is smoked, the high temperature will easily set the hydrated hydrochloric acid molecule free from the above mentioned salt [16] with the resulting acidification of the oral cavity.

The preparation of methamphetamine as a prescribed drug is well controlled and should have the minimum impurities, while the illegal preparation has only one goal and that is to make money with the minimum cost. Therefore, the quality of methamphetamine and impurities found on the street product at street level should vary a lot with possible side effects which are unknown.

Therefore, the purpose of this study was to determine the pH levels of different samples of methamphetamine on the street market in Cape Town.

## 2. Materials and Methods

Twenty-nine different samples of methamphetamine crystals used in the Cape Town Metropole were obtained. One mg of each methamphetamine sample was dissolved in 1.00 mL of distilled water and the pH measured using an Orion Expandable Ion Analyzer EA940 (Orion Research Inc., Mass, USA) and an Orion 9165BNWP Sure-Flow, Epoxy-body (Thermo Electron Corporation, Beverley, Mass, USA) at 25°C. Three determinations of each sample were done and the mean and median calculated.

## 3. Results

Table 1 gives the number of methamphetamine samples categorized in a specific pH range (stem and leave table). The median of the pH value was found to be equal to 4.87 and the average pH value equal to 5.0.

**Figure 1:**
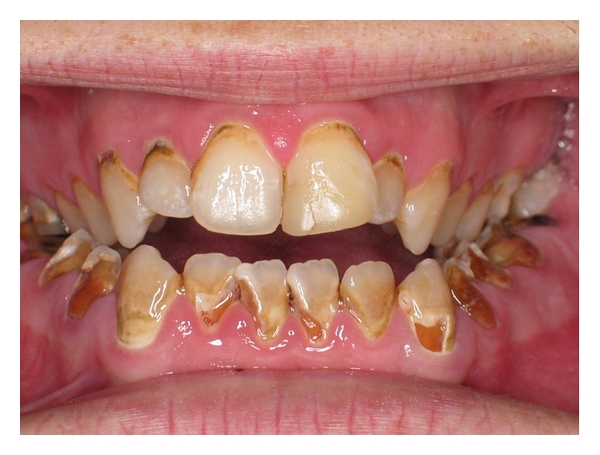
Close-up anterior view of maxillar and mandibular incisors of a 24-year-old Caucasian who has been using methamphetamine for 5 years.

Figure 1 gives a typical example of the anterior view of the maxillary and mandibular incisors of a methamphetamine user.

## 4. Discussion

The building blocks of human enamel (hydroxyapatite, fluorapatite) are mainly calcium, phosphate, hydroxy and fluoride ions. Thus, the solubility of enamel (dissolution) will mainly depend on the presence and concentrations of these ions as it will influence the degree of saturation to (relation between ion product and the solubility product) enamel. A variety of calcium phosphate salts are found in the oral cavity all of which will then have an influence on the solubility of enamel [17, 18]. The following are examples: brushite (CaHPO_4_·2H_2_O), octacalcium phosphate (Ca_8_(PO_4_)_4_(HPO_4_)_2_·5H_2_O), *β*-tricalcium phosphate (Ca_3_(PO_4_)_2_), fluorapatite (Ca_10_(PO_4_)_6_F_2_), and calcium fluoride (CaF_2_). When saliva is supersaturated with respect to a salt, it means that the salt will not dissolve or it will precipitate. 

Except from the above, it is also known that the pH (e.g., of the oral cavity) has a major effect on the solubility of enamel. In general, the lower (more acidic) the pH of the environment (oral cavity and saliva), the easier the enamel will dissolve. In a healthy mouth, where the saliva has a pH value close to neutrality (pH = 7.0), the saliva will always tend to buffer the oral cavity to a near neutral pH value which favors enamel. However, with the use of methamphetamine, the saliva secretion eventually stops (hyposalivation situation), the oral cavity becomes dry, and the buffering effect of saliva disappears with the result that anything acidic (like most methamphetamine samples) will have a higher dissolution effect on enamel and dentine.

Table 1 shows a high variation in the pH levels between the different methamphetamine samples. The pH values (Table 1) varied from 3.03 up to 7.03 with a median value of 4.87. Furthermore, 30% of the samples (9 of 29) had a low pH value of between 4.5 and 5.0, while 58.6% (17 of 29) of the samples had a pH value of between 4.0 and 5.0. Therefore, one could expect a very high variation in damage to teeth in the oral cavity due to the use of samples synthesized in different ways. In this sense, it can be foreseen that a methamphetamine sample with a pH value of 7.03 will not cause significant teeth dissolution (damage), while samples with lower pH values will be destructive to enamel with gradually increasing damage as the pH drops [19].

McCann [20] demonstrated that saliva is supersaturated with respect to hydroxyapatite and fluorapatite, close to saturation with respect to octacalcium phosphate and brushite, depending on the secretion rate, and invariably unsaturated with respect to calcium fluoride. Fluoride has various modes of action: enamel-containing fluoride (fluorapatite) is thousands of times less soluble than enamel without fluoride (hydroxyapatite). In the presence of fluoride, enamel demineralization starts at a lower pH. Furthermore, fluoride enhances the rate of crystal growth [21] which could result in larger crystallites and more resistant enamel and favors remineralization in the tooth decay-repair process. Fluoride also inhibits the plaque fermentation process and therefore the production of acid through plaque fermentation during the consumption of soft drinks high in sugars. A diagram by Ten Cate and Duijsters, 1983 [22], gives one a very good idea of the combined influence of pH, fluoride, and calcium on the solubility of enamel. In this graph, enamel dissolution was indicated by the free calcium concentration. From the graph it can be seen that there is an enormous increase in the solubility of enamel as the fluoride concentration decreases from 5 ppm to 0 ppm and when the pH decreases from 5 to 4. This indicates what could happen to enamel under the influence of an acidic methamphetamine sample especially in the absence of saliva flow as a result of the use of the drug. When the saliva flow is normal, one should expect less damage to enamel during the oral use of methamphetamine because the saliva has a buffering capacity which will tend to neutralize the acid from the sample. Furthermore, saliva may also contain fluoride and calcium ions which could have a protecting or even a remineralizing effect on damaged enamel. It is also possible to remineralize damaged enamel by drinking milk [23], eating hard cheese [24], or rinsing with fluoride-containing solutions.

The photo is that of a 24-year-old Caucasian male attended the outpatient clinic at the Faculty of Dentistry, University of the Western Cape, complaining of severe pain in his left lower posterior teeth. The patient presented with severe dental pain, bad breath, and self-reported poor dental appearance. He reported using methamphetamine for five years and had not experienced much caries prior to using the drug. This scenario gives one a practical example of what the drug can do to teeth of people.

Therefore, the following are a number of ways one could practice to minimize the enamel damaging effect through the use of methamphetamine:

- do not use methamphetamine;
- exercise good oral hygiene which include flossing and brushing;
- when thirsty drink artificially sweetened drinks or better drink milk and eat cheese;
- rinse with a fluoride containing mouth rinse.

## Figures and Tables

**Table 1:** The number of methamphetamine samples categorized in a specific pH range (stem and leave table) and the median pH.

pH values	No. of samples in area
3.0–3.4	2
3.5–3.9	2
4.0–4.4	4
4.5–4.9	9
5.0–5.4	4
5.5–5.9	2
6.0–6.4	3
6.5–6.9	1
7.0–7.4	2

Median pH = 4.87	
